# Preoperative CT-based radiomics nomogram to predict the micropapillary pattern in lung adenocarcinoma of size 2 cm or less

**DOI:** 10.3389/fonc.2024.1426284

**Published:** 2025-01-07

**Authors:** Xiaoyu Pan, Liang Fu, Jiecai Lv, Lijuan Feng, Kai Li, Siqi Chen, Xi Deng, Liling Long

**Affiliations:** ^1^ Department of Radiology, The First Affiliated Hospital of Guangxi Medical University, Nanning, Guangxi, China; ^2^ Department of Radiology, The Second Affiliated Hospital of Guangxi Medical University, Nanning, Guangxi, China

**Keywords:** lung adenocarcinoma, micropapillary pattern, radiomics, nomogram, computed tomography

## Abstract

**Purpose:**

To develop and validate a radiomics nomogram model for predicting the micropapillary pattern (MPP) in lung adenocarcinoma (LUAD) tumors of ≤2 cm in size.

**Methods:**

In this study, 300 LUAD patients from our institution were randomly divided into the training cohort (n = 210) and an internal validation cohort (n = 90) at a ratio of 7:3, besides, we selected 65 patients from another hospital as the external validation cohort. The region of interest of the tumor was delineated on the computed tomography (CT) images, and radiomics features were extracted. A nomogram model was established using radiomics features, clinical features and conventional radiographic features. The nomogram model was compared with the radiomics model and the clinical model alone to test its diagnostic validity. Receiver operating characteristic (ROC) curves, areas under the ROC curves and decision curve analysis (DCA) results were plotted to evaluate the model performance and clinical application.

**Results:**

The nomogram model exhibited superior performance, with an AUC of 0.905 (95% confidence interval [CI]: 0.857-0.951) in the training cohort, which decreased to 0.817 (95% CI: 0.698-0.936) in the external validation cohort. The clinical model had AUCs of 0.820 (95% CI: 0.753-0.886) and 0.730 (95% CI: 0.572-0.888) in the training and external validation cohorts, respectively. The radiomics model had AUCs of 0.895 (95% CI: 0.840-0.949) and 0.800 (95% CI: 0.675-0.924) for training and external validation, respectively. DCA confirmed that the nomogram model had the better clinical benefit.

**Conclusions:**

The nomogram model achieved promising prediction efficiency for identifying the presence of the MPP in LUAD tumors ≤2 cm, allowing clinicians to develop more rational and efficacious personalized treatment strategies.

## Introduction

Lung cancer is the primary cause of cancer-related mortality worldwide (1). Among the various pathological types of non-small cell lung cancer (NSCLC), lung adenocarcinoma (LUAD) has emerged as the most prevalent (2). In 2015, the World Health Organization (WHO) categorized LUAD into five primary histologic subtypes delineated by prognosis (3). Prior studies have indicated that the micropapillary pattern (MPP) is classified as the high-risk subtype owing to its significant correlation with factors indicating adverse prognosis, including tumor spread through air spaces (4), lymph node metastasis (5), and vascular invasion (6). Wang et al. demonstrated that among LUAD patients with pathological stage pT1N0M0, those with the MPP and solid pattern subtypes exhibited poorer recurrence-free survival (RFS) and overall survival (OS) than patients with the other subtypes (7). Notably, the MPP represents less than 5% of the tumor volume but still negatively affects OS (8).

The advancement of imaging technology and the widespread adoption of low-dose computed tomography (CT) have led to a significant increase in the detection of early-stage lung cancers. In patients with resectable NSCLC, lobectomy is widely acknowledged as the standard surgical procedure (9). With the ongoing advancements in thoracic surgical, sublobar resection, including segmentectomy and wedge resection, have become increasingly prevalent in the treatment of NSCLC with a diameter of 2 cm or less. In two Phase III trials involving patients with tumor sizes of 2 cm or smaller, sublobar resection demonstrated superiority or non-inferiority to lobectomy regarding disease-free and overall survival (10, 11). However, Xu et al. recommended that LUAD patients with a tumor size ≤2 cm and the MPP constituting more than 5% of tumor volume should undergo lobectomy and systematic lymph node dissection (12). Given the unfavorable prognosis linked to MPP, sublobar resections may be inappropriate for this patient population. Therefore, the preoperative diagnosis of MPP in LUAD with a tumor size ≤2 cm has significant implications for the choice of surgical procedure.

Rapid advancements in artificial intelligence are transforming imaging medicine from a basic diagnostic tool into an essential element of personalized precision medicine. Radiomics is a technique that enables the noninvasive and quantitative description of the biological characteristics and heterogeneity of a tumor (13). Previous studies have demonstrated the utility of radiomics in predicting the MPP and solid subtypes of LUAD (14–17). Previous studies included patients with tumor diameters greater than 2 cm, radiomics models developed without accounting for tumor diameter may not be suitable for identifying MPP in LUAD patients with tumor size ≤2 cm. In addition, the significance of radiographic features in the construction of predictive models has been neglected, and radiographic features may enhance the diagnostic efficacy of the model. Furthermore, several studies failed to perform external validation.

Therefore, in this study, we aimed to develop and validate a machine learning model by CT radiomics signature with clinical features and conventional radiographic features to predict the MPP in LUAD tumors ≤2 cm in size.

## Materials and methods

The study followed the Radiomics Quality Score (RQS) system. The RQS scoring criteria, the scores for this study, and the rationale underlying for the scores for prediction model development are detailed in the **Supplementary Material.**

### Patients and data acquisition

**Figure 1:**
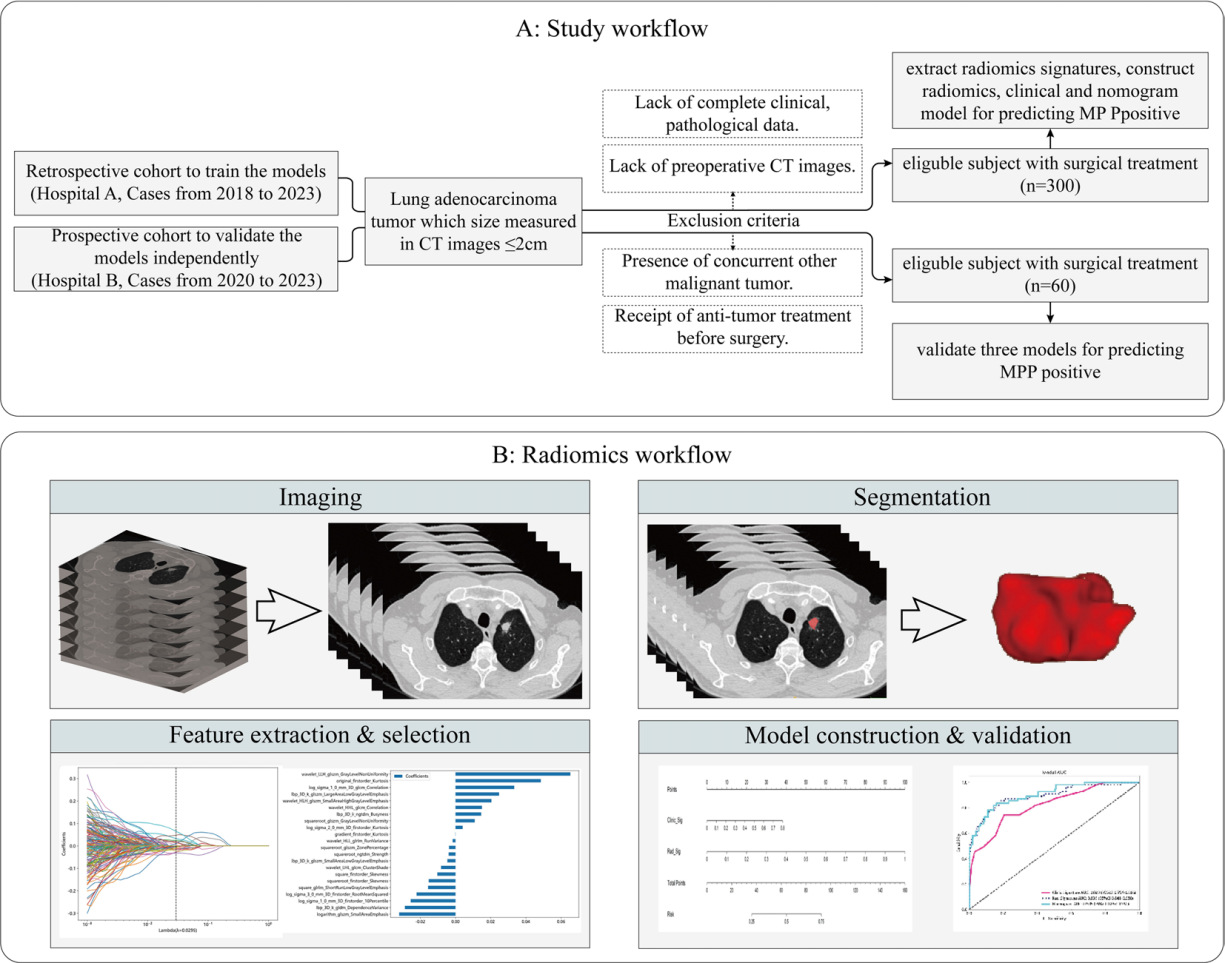
Flowchart of the study subjects. Study workflow **(A)**. Radiomics workflow **(B)**. MMP, Micropapillary Patter; CT, computed tomography.

This retrospective study was approved by the Institutional Ethics Committee of the First Affiliated Hospital of Guangxi Medical University and the Institutional Ethics Committee of the Second Affiliated Hospital of Guangxi Medical University, and the requirement for patient informed consent was waived(2024-E246-01, 2024-KY (0780). We selected patients who were treated at the First Affiliated Hospital of Guangxi Medical University (hospital A) from January 2018 to September 2023 as the training and internal validation cohorts. In addition, the patients for the external validation cohort were obtained from the Second Affiliated Hospital of Guangxi Medical University (hospital B). The study flowchart is shown in **Figure 1A**. The inclusion criteria were as follows: 1. All enrolled patients underwent chest CT scans within two weeks before surgery, and the maximum diameter of the tumor on CT images was ≤2 cm. 2. Pathology confirmed primary LUAD. 3. Patients did not receive radiotherapy, chemotherapy, or immunotherapy before surgery. 4. There was no history of other malignant tumors. The exclusion criteria were as follows: 1. Lack of complete clinical and pathological data and preoperative CT images. 2. Presence of other concurrent malignant tumors. 3. Receipt of anti-tumor treatment before surgery. 4. Presence of multiple pulmonary tumor lesions. 5. patients with lung fibrosis, COPD, or prior lung resections.

**Figure 2:**
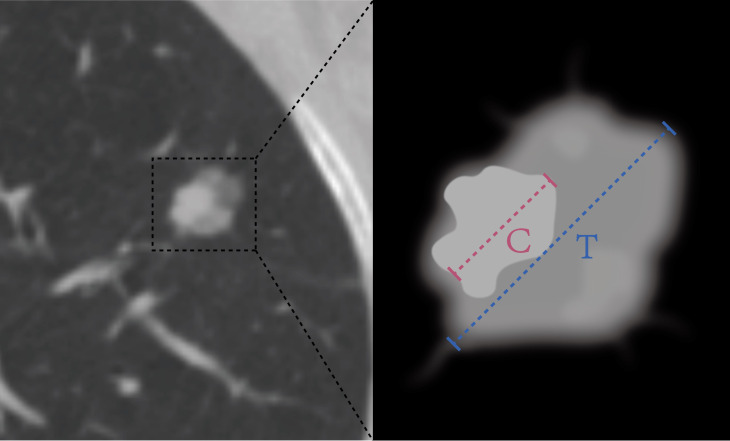
The HRCT imaging information measurement contains the following: (1) the diameter of the tumor (T) was defined as the largest axial diameter of the lesion on the lung window setting (Blue dotted line); (2) the diameter of consolidation (C) on the axial image on the lung window setting was measured, and consolidation was defined as an area of increased opacification that completely obscured the underlying bronchial structures and vascular markings (Pink dotted line); (3) the ratio of the maximum diameter of consolidation relative to the maximum tumor diameter from the lung window (CTR).

**Figure 3:**
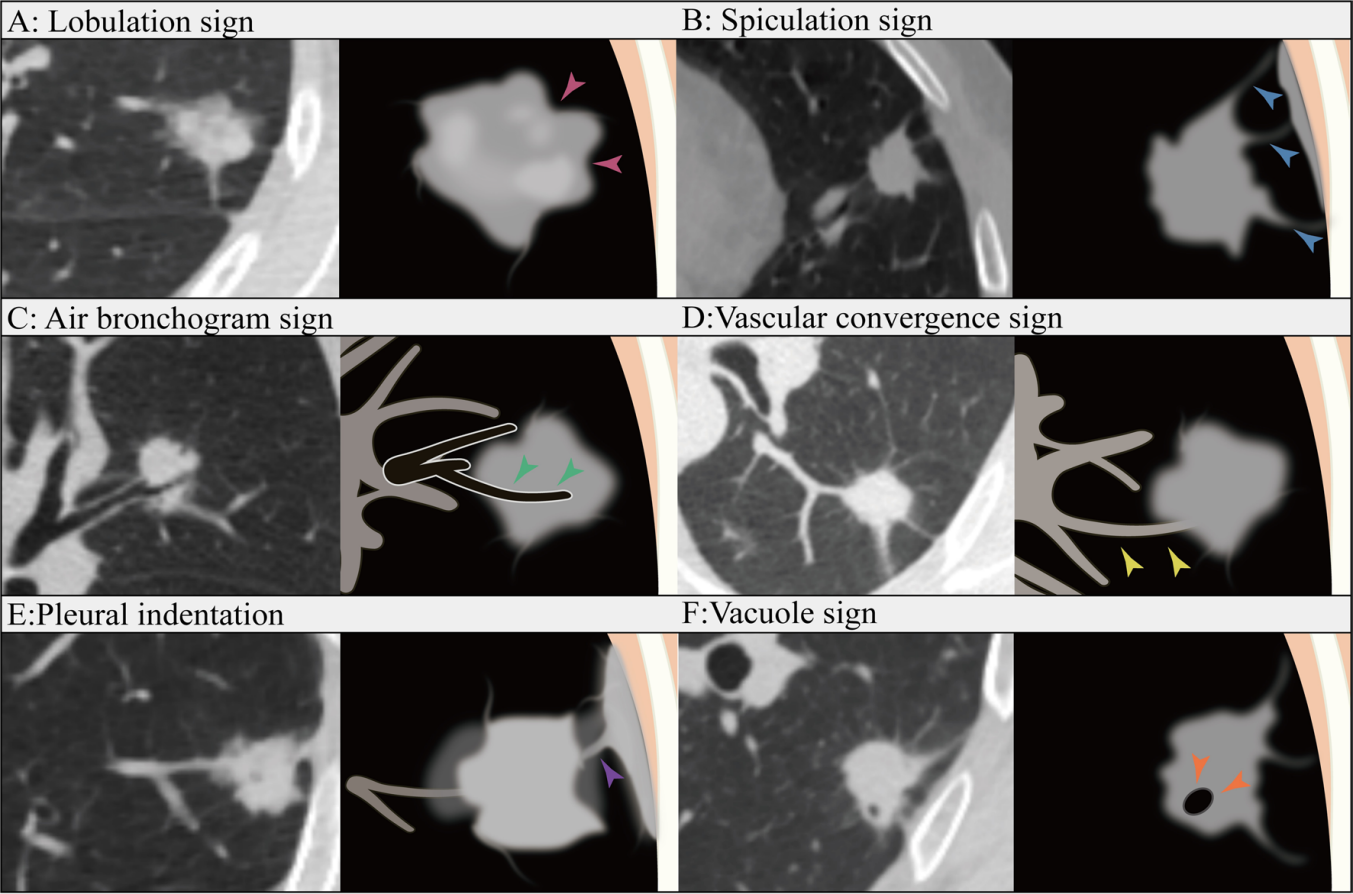
Lobulation sign **(A)**: The contour of the tumor appear as an arched protrusion, with alternating arcs creating a lobulated appearance, referred to as the lobulation sign (pink arrows). Spiculation sign **(B)**: The edges of the tumor display varying degrees of spiculated projections, commonly referred to as the Spiculation sign (blue arrows). Air bronchogram sign **(C)**: The tumor contains bronchial structures that are filled with air (green arrows). Vascular convergence sign **(D)**: The vascular convergence sign refers to the phenomenon in which blood vessels converge towards a particular lesion in certain diseases or pathological conditions as observed in imaging (yellow arrows). Pleural indentation **(E)**: Pleural indentation refers to the presence of indentations or impressions on the pleura (purple arrow). Vacuole sign **(F)**: The vacuole sign refers to the presence of low-density areas within a tumor, typically measuring between 1 mm and 3 mm in diameter(orange arrows).

In total, our study included patients with a total of 365 lesions that were diagnosed as LUAD. Among them, 300 patients from the First Affiliated Hospital of Guangxi Medical University were randomly divided into a training cohort (n=210) and an internal validation cohort (n=90) at a ratio of 7:3. Additionally, 65 cases from the Second Affiliated Hospital of Guangxi Medical University were recruited as the external validation cohort. Clinical data and conventional radiographic features, including age, sex, smoking history, preoperative carcinoembryonic antigen (CEA) level, maximum tumor diameter, vascular convergence sign, nodule type(part solid nodule and solid nodule), lobulation sign, spiculation sign, vacuole sign, air bronchogram sign, pleural indentation sign, and CTR, were collected. The maximum tumor diameter was defined as the maximum diameter on the axial plane in the lung window, and the solid tumor size was defined as the maximum diameter of the solid component. The CTR was calculated as the ratio of the solid tumor size over the tumor size (**Figure 2**). We have added figures to explain signs like lobulation sign, spiculation sign, air bronchogram sign, vascular convergence sign, pleural indentation, vacuole sign. The figures were detailed in the **Figure 3**.

### CT imaging

For CT imaging, the following scanners were used: GE256 row Revolution CT, Siemens Definition Flash CT, Siemens Dual-source CT, Siemens Force CT, and Philips iCT 128. The scanning parameters were as follows: tube voltage, 120 kV; tube current, 250-300 mA; matrix, 512×512; and reconstructed slice thicknesses, 0.6 mm~1.5 mm. The window settings were adjusted to a lung window width of 1500 HU and a window level of -500 HU.

### Histologic evaluation

Two experienced pathologists independently assessed the histological subtypes according to the 2015 WHO classification of LUAD (3), resolving any disagreements through discussion to reach a consensus. In our study, all patients were divided into two groups based on the proportion of MPP in the tumor, the positive group included patients with ≥5% MPP, while the negative group included patients with <5% MPP and patients without MPP (18).

### Lesion segmentation and radiomics feature extraction

The radiomics analysis workflow involved lesion segmentation, feature extraction, feature selection, and model construction (**Figure 1B**). All lesions were detected on the CT images. Regions of interest (ROIs) were manually segmented slice by slice along the lesions using open software (ITK-SNAP 3.8.0 available at www.itksnap.org) by 2 radiologists from hospital A who were blinded to the patients’ histologic information. The segmentations of X.P with 7 years of diagnostic experience and L.F with 9 years of diagnostic experience were compared for interobserver differences. Repeated measurements were performed at an interval of 2 weeks, and the segmentations were compared for intraobserver differences. The intraobserver and interobserver differences were assessed by calculating the intraclass correlation coefficient (ICC) and features with consistency values<0.7 were removed. Finally, radiomics features were extracted using the ROI of the first segmentation of the radiologist with 9 years of diagnostic experience. Prior to segmentation, we standardized the CT images and resampled them to voxel sizes of 1 mm × 1 mm × 1 mm. The extracted features were divided into the following categories: first-order, grey-level co-occurrence matrix (GLCM), grey-level dependence matrix (GLDM), grey-level size-zone matrix (GLSZM), grey-level run length matrix (GLRLM), and neighboring grey tone difference matrix (NGTDM). In total, 1835 radiomics features were extracted using pyradiomics (version 2.2.0) from each ROI.

### Feature selection and radiomics model construction

We performed the Mann−Whitney U test for statistical analysis and feature screening on all radiomics features, retaining only those with *p* values<0.01. Features demonstrating high repeatability were subjected to Spearman’s rank correlation coefficient analysis to assess correlations between features. Features with a correlation coefficient >0.9 between any two features were retained. To preserve the comprehensive depiction of features, a greedy recursive deletion strategy was employed for feature filtering, wherein the most redundant feature in the current set was iteratively removed. Then, the least absolute shrinkage and selection operator (LASSO) regression model was subsequently applied to the discovery dataset to construct the signature. Depending on the regularization weight λ, LASSO shrinks all regression coefficients towards zero and assigns coefficients of irrelevant features to zero. To determine the optimal λ, 10-fold cross-validation with minimum criteria was utilized, where the final λ value was chosen based on the minimum cross-validation error. The features with nonzero coefficients were retained for regression model fitting and amalgamated into a radiomics signature. Subsequently, a radiomics score was computed for each patient through a linear combination of retained features weighted by their model coefficients. The Python scikit-learn package was used for LASSO regression modelling. After feature screening, 22 features were input into the logistic regression (LR) model for risk model construction. Here, we adopted 10-fold cross-validation to obtain the final radiomics signature.

### Construction of the clinical model and nomogram

The process used to construct the clinical model was almost the same as that used for the radiomics model. First, univariable analysis was performed on clinical indicators and CT morphological features to identify factors with a *p*-value < 0.05. Subsequently, multivariable logistic regression was used to determine the independent factors associated with MPP. We also used the same machine learning model in the radiomics model-building process. The nomogram model was developed by integrating the radiomics signature with the clinical independent factors. In every cohort, Receiver operating characteristic (ROC) curves were used to assess the diagnostic performance of models to identify the presence of MPP in LUAD. The AUCs of the models were evaluated with the Delong test. The calibration curves were used to assess the concordance between the predictions of the nomogram and the actual observations. Decision curve analysis (DCA) was utilized to map and evaluate the clinical utility of the predictive models.

### Statistical analysis

Statistical analyses were performed using SPSS (version 26.0; IBM Corp.) and the “One-key AI” platform (https://www.medai.icu), which is based on PyTorch 1.8.0. Normally distributed data were analyzed using independent t tests, and nonnormally distributed data are expressed as medians (interquartile ranges) and analyzed using Mann−Whitney *U* tests. Categorical variables were analyzed using chi-square tests. The independent predictors of MPP in LUAD patients were determined by univariable and multivariable logistic regression analyses. Bilateral *p* values < 0.05 were considered to indicate statistical significance. ROC curves were drawn, and the area under the curve (AUC) was calculated to assess the diagnostic performance of each model. The AUCs of the models were evaluated with the Delong test. DCA was utilized to map and evaluate the clinical utility of the predictive models.

## Results

### Clinical characteristics

The clinical factors and conventional radiographic characteristics of the patients are listed in **Table 1**. In the training cohort, there was a statistically significant difference (*P* < 0.05) in maximum tumor diameter, sex, nodule type, vascular convergence sign, and CTR between the MPP-positive and MPP-negative groups. The maximum tumor diameter was greater in the MPP-positive group (1.72 ± 0.23 cm vs. 1.50 ± 0.24 cm, p<0.001). The solid type was the predominant type in the MPP-positive group (69.1% vs. 30.1%, p<0.001). In the training cohort, internal validation cohort and external validation cohort, the variables age, smoking history, CEA level, lobulation sign, spiculation sign, vacuole sign, air bronchus sign and plural indentation did not significantly differ between the MPP-positive and MPP-negative groups (P>0.05). Nodule type and maximum tumor diameter were identified as the independent predictors of the MPP in LUAD patients through multivariable logistic regression analysis (**Table 2**).

**Table 1:** Clinical and conventional radiographic features of patients in the training and two validation cohorts.

Characteristic	Training cohort (n=210)			Internal validation cohort (n=90)			External validation cohort (n=65)		
	MPP-positive(n=55)	MPP-negative(n=155)	*p*	MPP-positive(n=28)	MPP-negative(n=62)	*p*	MPP-positive(n=17)	MPP-negative(n=48)	*p*
Gender			0.039			1			1
Male	27(49.10%)	50(32.3%)		12(42.9%)	26(41.9%)		7(41.2%)	19(39.6%)	
Female	28(50.9%)	105(67.7%)		16(57.1%)	36(58.1%)		10(58.8%)	29(60.4%)	
Age, years	60.22 ± 11.39	57.52 ± 8.43	0.065	62.32 ± 10.94	59.24 ± 9.11	0.167	56.24 ± 10.11	57.96 ± 10.07	0.547
Smoking history			0.142			0.742			0.086
Yes	17(30.9%)	31(20%)		7(25.0%)	12(19.4%)		6(35.3%)	6(12.5%)	
No	38(69.1%)	124(80%)		21(75.0%)	50(80.6%)		11(64.7%)	42(87.5%)	
CEA			0.076			0.418			0.594
Yes	10(18.2%)	14(9.0%)		4(14.3%)	4(6.5%)		2(4.2%)	2(11.8%)	
No	45(81.8%)	141(91.0%)		24(85.7%)	58(93.5%)		46(95.8%)	15(88.2%)	
Maximum tumor diameter,cm	1.72 ± 0.23	1.50 ± 0.24	<0.001	1.61 ± 0.25	1.49 ± 0.25	0.027	1.65 ± 0.31	1.57 ± 0.27	0.367
Nodule type			<0.001			<0.001			0.003
SN	38(69.1%)	44(28.4%)		23(82.1%)	22(35.5%)		13(76.5%)	15(31.3%)	
PSN	17(30.9%)	111(71.6%)		5(17.9%)	40(64.5%)		4(23.5%)	33(68.7%)	
Vascular convergence sign			<0.001			1			0.321
Yes	20(36.4%)	19(12.3%)		9(32.1%)	21(33.9%)		5(29.4%)	7(14.6%)	
No	35(63.6%)	136(87.7%)		19(67.9%)	41(66.1%)		12(70.6%)	41(85.4%)	
Spiculation sign			0.052			0.588			0.711
Yes	36(65.5%)	76(49.0%)		16(57.1%)	30(48.4%)		9(52.9%)	21(43.8%)	
No	19(34.5%)	79(51.0%)		12(42.9%)	32(51.6%)		8(47.1%)	27(56.2%)	
Lobulation sign			0.770			0.955			0.297
Yes	54(98.2%)	149(96.1%)		27(96.4%)	58(93.5%)		17(100.0%)	42(87.5%)	
No	1(1.8%)	6(3.9%)		1(3.6%)	4(6.5%)		0(0%)	6(12.5%)	
Vacuole sign			0.565			0.816			0.438
Yes	68(43.9%)	21(38.2%)		10(35.7%)	19(30.7%)		5(29.4%)	8(16.7%)	
No	87(56.1%)	34(61.8%)		18(64.3%)	43(69.3%)		12(70.6%)	40(83.3%)	
Air bronchogram			1			1			0.499
Yes	14(25.5%)	39(25.2%)		5(17.9%)	11(17.7%)		6(35.3%)	11(22.9%)	
No	41(74.5%)	116(74.8%)		23(82.1%)	51(82.3%)		11(64.7%)	37(77.1%)	
Plural indentation			0.209			0.082			0.502
Yes	32(58.2%)	73(47.1%)		18(64.3%)	26(41.9%)		9(52.9%)	19(39.6%)	
No	23(41.8%)	82(52.9%)		10(35.7%)	36(58.1%)		8(47.1%)	29(60.4%)	
CTR			<0.001			0.001			0.095
<50%	3(5.5%)	61(39.4%)		0(0%)	21(33.9%)		2(11.8%)	18(37.5%)	
≥50%	52(94.5%)	94(60.7%)		28(100.00)	41(66.1%)		15(88.2%)	30(62.5%)	

Unless otherwise specified, categorical variables are presented as number (%).

MPP, Micropapillary Patter**;** SN,Solid Nodule**;**PSN,Part Solid Nodule.

**Table 2:** Univariable and multivariable logistic regression analysis of Characteristic in the training cohorts.

Characteristic	Univariable logistic regression		Multivariable logistic regression	
	OR (95% CI)	*p* value	OR (95% CI)	*p* value
Gender	0.869(0.784-0.964)	0.026	0.92(0.841-1.006)	0.125
Vacuole sign	0.956(0.863-1.059)	0.466		
Age	1.006(1.001-1.011)	0.066		
Air bronchogram	1.003(0.893-1.126)	0.066		
Spiculation sign	1.136(1.028-1.255)	0.036	0.918(0.834-1.01)	0.142
Plural indentation	1.089(0.985-1.204)	0.159		
Smoking history	1.127(1.00-1.27)	0.099		
Lobulation sign	1.131(0.855-1.496)	0.469		
CEA	1.191(1.018-1.394)	0.067		
Vascular convergence sign	1.361(1.202-1.542)	<0.001	1.133(1.004-1.278)	0.089
CTR	1.362(1.229-1.511)	<0.001	1.135(1.014-1.27)	0.064
Nodule type	1.392(1.265-1.533)	<0.001	1.288(1.157-1.432)	<0.001
Maximum tumor diameter	1.914(1.598-2.291)	<0.001	1.819(1.539-2.149)	<0.001

OR, odds ratio; CI, confidence interval.

### Feature selection and Rad score establishment

**Figure 4:**
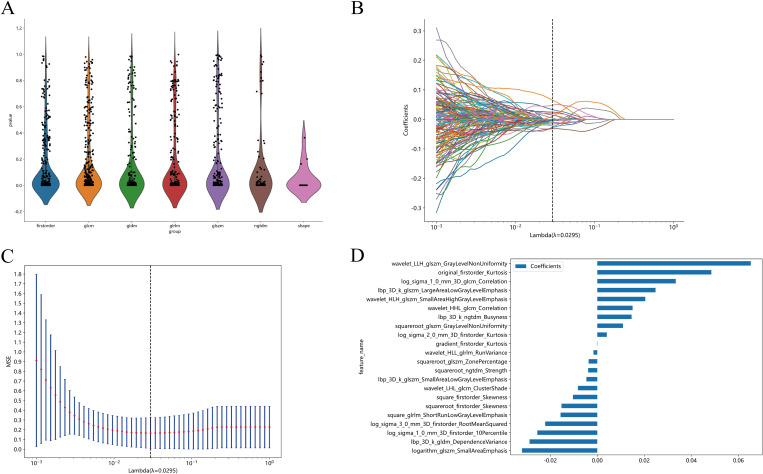
All the features and the corresponding *p*-value results **(A)**. Radiomics feature selection based on LASSO algorithm and Rad score establishment **(B)**; Ten-fold cross-validated coefficients and 10-fold cross-validated MSE **(C)**;The histogram of the Rad score based on the selected features **(D)**.

**Figure 5:**
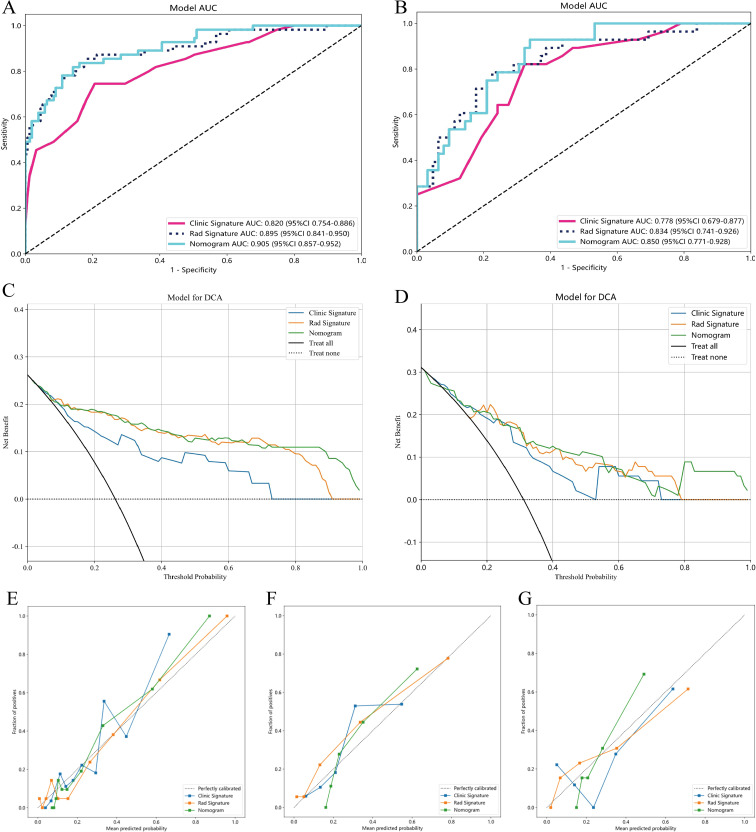
AUC Comparison of clinical, Radiomics, and nomogram models in the training cohort **(A)** and internal validation cohort **(B)**;Decision curves of the clinical, radiomic, and nomogram models in the training and internal validation cohort **(C, D)**, The combined nomogram performed optimally in both the training and internal validation cohort. The calibration curves in three cohorts: training cohort **(E)**, internal validation cohort **(F)** and external validation cohort **(G)**, calibration curves are presented based on three models for predicting the micropapillary components of LUAD. The x-axis represents the predicted micropapillary components probabilities based on the clinic signature, radiomics signature, and nomogram. At the same time, displays the actual probabilities of these components. The 45° diagonal line symbolizes the ideal prediction, with the blue, yellow, and green lines representing the predictive performance of the clinical, radiomics, and nomogram, respectively.

**Figure 6:**
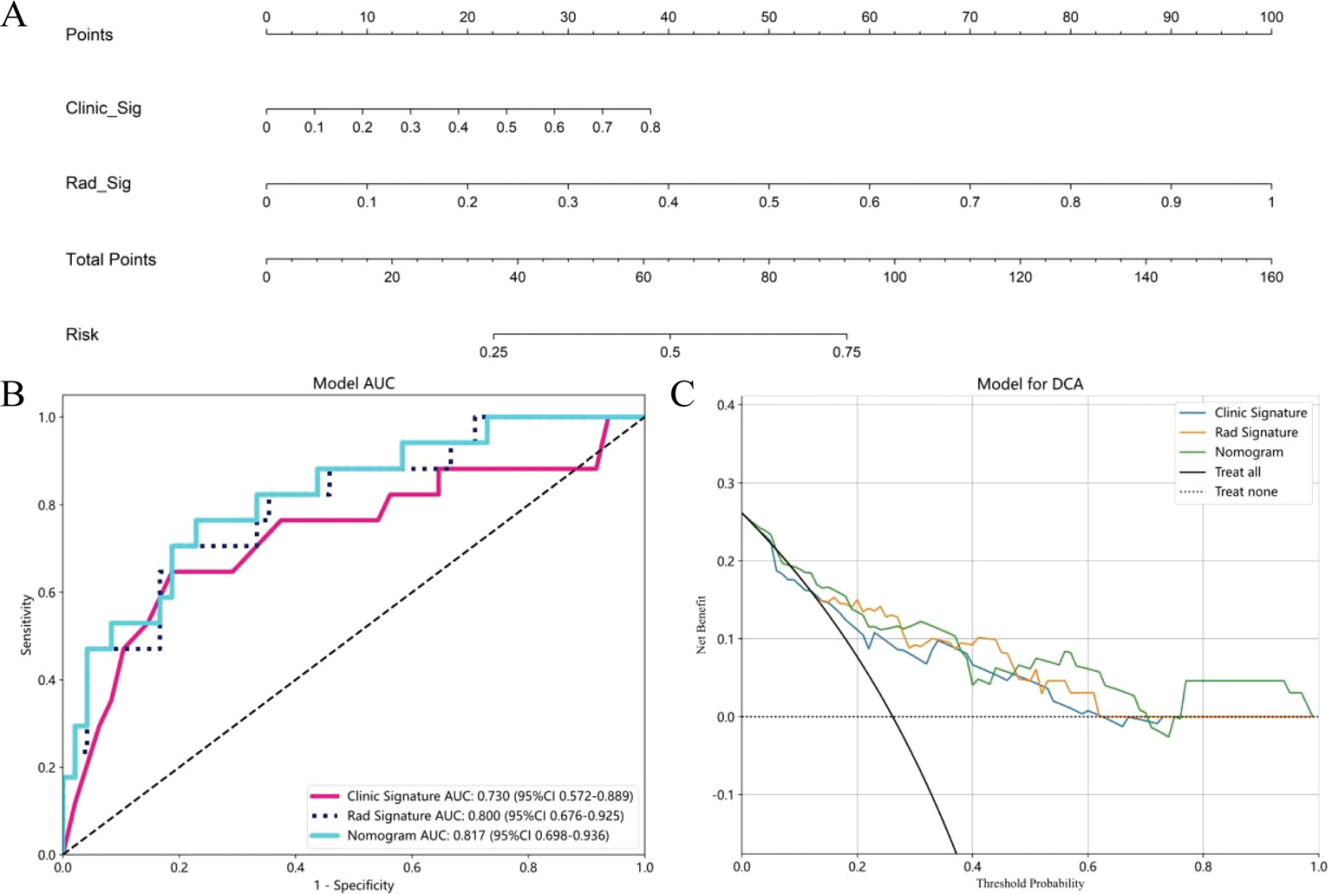
The nomogram to distinguishing MPP in LUAD with size ≤ 2cm, Clinic Sig, Clinical Signature; Rad Sig, Radiomics Signature **(A)**; AUC Comparison of clinical, Radiomics, and nomogram models in the external validation cohort **(B)**; Decision curves of the clinical, radiomics, and nomogram models in the external validation cohort **(C)**.

After selection, a total of 22 features with a nonzero coefficient value remained. The radiomics signature was constructed based on the coefficient values of the selected features. The details of the features are shown in **Figure 4**. The radiomics model was constructed by using all selected features. In the training cohort, the model had an AUC of 0.895 (95% CI 0.840-0.949) with balanced sensitivity and specificity of 0.836 and 0.819, respectively. In the internal validation cohort, the AUC was 0.834 (95% CI 0.741-0.925), with a sensitivity and specificity of 0.750 and 0.774, respectively (**Table 3**, **Figures 5A, B**). In the external validation cohort, the AUC was 0.800 (95% CI 0.675-0.924), and the sensitivity and specificity were 0.647 and 0.812, respectively (**Table 3**, **Figure 6B**).

**Table 3:** The performance of three models in training and two validation cohorts.

Model	Cohort	AUC(95% CI)	Accuracy	Sensitivity	Specificity
Radiomics	Training cohort	0.895(0.840-0.949)	0.824	0.836	0.819
	Internal validation cohort	0.834(0.741-0.925)	0.767	0.750	0.774
	External validation cohort	0.800(0.675-0.924)	0.769	0.647	0.812
Clinical	Training cohort	0.820(0.753-0.886)	0.781	0.673	0.819
	Internal validation cohort	0.778(0.678-0.877)	0.700	0.643	0.726
	External validation cohort	0.730(0.572-0.888)	0.769	0.529	0.854
Nomogram	Training cohort	0.905(0.857-0.951)	0.843	0.800	0.858
	Internal validation cohort	0.850(0.770-0.928)	0.733	0.893	0.661
	External validation cohort	0.817(0.698-0.936)	0.754	0.706	0.771

AUC, area under curve; CI, confidence interval.

### Establishment and performance of the clinical model and nomogram model

Multivariate logistic regression analysis revealed that nodule type and maximum tumor diameter were independent predictors of the presence of MPP in LUAD patients, and these features were used to establish the clinical model. In the training cohort, this model had an AUC of 0.820 (95% CI 0.753-0.886) with a sensitivity and specificity of 0.673 and 0.819, respectively. In the internal validation cohort, the AUC was 0.778 (95% CI 0.678-0.877), with a sensitivity and specificity of 0.643 and 0.726, respectively (**Table 3**, **Figures 5A, B**). A nomogram was constructed based on the radiomics-clinical model (**Figure 5A**). In the training cohort, the nomogram model had an AUC of 0.905 (95% CI 0.857–0.951), with a sensitivity and specificity of 0.800 and 0.858, respectively. In the internal validation cohort, the AUC was 0.850 (95% CI 0.770–0.928), and the sensitivity and specificity were 0.893 and 0.661, respectively (**Table 3**, **Figures 5A, B**). In the external validation cohort, the AUC of the clinical model was 0.730 (95% CI 0.675-0.924); the sensitivity and specificity were 0.529 and 0.854, respectively; the AUC of the nomogram model was 0.817 (95% CI 0.698-0.936); and the sensitivity and specificity were 0.706 and 0.771, respectively (**Table 3**, **Figure 6B**). DCA was employed to assess the clinical application of the three developed models. The DCA results indicated that the nomogram model demonstrated the better clinical benefit in distinguishing patients with MPP from those without MPP (**Figures 5C, D**, **6C**). The nomogram to distinguishing MPP in LUAD with size ≤ 2cm (**Figure 6A**). In order to compare the clinical model, radiomics model and nomogram, the AUCs of the models were evaluated with the Delong test (**Supplementary Figure 1**, **Supplementary Table 1**). The AUCs of the nomogram, radiomics in the training cohorts were significantly different from the clinical model (*p*<0.05). The calibration curves in three cohorts, training cohort (**Figure 5E**), internal validation cohort (**Figure 5F**) and external validation cohort (**Figure 5G**) indicated good agreement between predicted probability and actual occurrence.

## Discussion

The incidence and mortality of lung cancer, which were already high, have increased globally. In 2015, China ranked first globally in terms of both incidence (11.4%) and mortality (18%) of lung cancer (19). An estimated 609,820 people in the United States died from cancer in 2023, and the greatest number of deaths were from lung cancer (1).Surgical resection is currently the main treatment for NSCLC, although some scholars suggest that segmentectomy should be the standard surgical procedure for treating NSCLC lesions that are ≤2 cm in size (20). Notably, the presence of MPP accounting for more than 5% of the total tumor volume is an independent risk factor for recurrence and poor outcomes in lung LUAD with a size of ≤ 2 cm. This suggests that wedge resection or segmentectomy may not represent the optimal surgical procedure for these patients. Therefore, this study was aimed to develop predictive models, including a clinical model, a radiomics model, and a nomogram, to estimate MPP in patients with LUAD. The results of this research demonstrated the ability of the three developed predictive models to accurately identify MPP in LUAD patients with a tumor size ≤2 cm.

We developed a clinical model to identify MPP in LUAD patients, utilizing the clinical features and conventional radiographic features of 365 patients recruited from two hospitals. The reported incidence of MPP positivity in LUAD with a diameter of ≤2 cm varies across different studies (21, 22). The incidence of MPP is correlated with the solid component of the tumor. The two studies have incorporated pure ground glass opacity (pGGO), while our study did not include it. This distinction may account for the higher incidence rate observed in our findings compared to those reported in other studies. By employing multivariable logistic regression analysis, we identified nodule type and maximum tumor diameter as independent predictors. Therefore, we used two indicators, nodule type and maximum tumor diameter, to construct a clinical model. The performance of the clinical model demonstrated promising discriminatory power, with an AUC of 0.820 in the training cohort, 0.778 in the internal validation cohort, and 0.730 in the external validation cohort. In the training and internal validation cohort, significant differences in nodule type, maximum tumor diameter and CTR were found between the MPP-positive group and the MPP-negative group, with the MPP-positive patients having larger diameters, a greater number of solid nodules and more solid components than did the patients in the negative group. Previous studies have recognized the CTR as a significant predictor of aggressiveness in LUAD (23). The CTR is also instrumental in differentiating various subtypes of adenocarcinoma. Xu observed that lung adenocarcinomas with a CTR exceeding 0.5 were more likely to exhibit MPP (24).Chen et al. (25) employed the CTR as the main morphological predictor to develop a predictive model for micropapillary/solid components in LUAD, achieving an AUC of 0.85 and demonstrating comparable predictive power to that of radiomics models. A study by Wang et al. (26)found that the maximum tumor diameter serves as a valuable indicator for differentiating between MPP groups, with those MPP positive group displaying a significantly larger maximum tumor diameter. This observation aligns with the findings presented in our study. The larger the diameter of the solid nodule is, the greater the aggressiveness of the tumor cells, and the greater the probability of the presence of high-grade components. In summary, Our study have demonstrated that the maximum tumor diameter, nodule type, and CTR all contribute to distinguishing MPP in LUAD patients with a tumor size ≤2 cm, further research and model development are necessary to improve its diagnostic accuracy. Our study also revealed that conventional radiographic features, including lobulation signs, spiculation signs, pleural indentation, vascular convergence signs, vacuole signs, and air bronchograms, did not significantly differ between the MPP-positive group and the MPP-negative group. This finding deviates from those of earlier studies (26–28), suggesting potential inconsistencies or limitations in the current understanding of the diagnostic utility of these features. The observed correlation between conventional radiographic features and the MPP status in this study could be influenced by the smaller tumor sizes, warranting additional research with a larger and more diverse sample to account for potential size-related variations. Subjectivity and the inherent difficulty in quantifying conventional radiographic features contribute to their potential limitations as diagnostic tools, as they might not consistently capture the heterogeneity of tumor cells. As a result, predictive models that solely rely on clinical parameters and conventional radiographic features may fail to fully meet the diagnostic needs for MPP in patients with LUAD.

Radiomics, a multidisciplinary approach that extracts high-dimensional data from medical images, has gained substantial recognition for its utility in diagnosing and evaluating the prognosis of lung diseases (29, 30). Previous research has established the usefulness of radiomics in predicting MPP in patients with LUAD. However, inconsistencies and substantial disparities exist among the findings of prior studies. In a previous study, researchers (31) employed a primary cohort of 286 patients and an external validation cohort of 193 patients to develop the radiomics model, achieving internal validation and external validation AUCs of 0.75 and 0.70, respectively. However, this multicentre studies demonstrated limited diagnostic performance, warranting further refinement, and their models lacked the integration of clinical data and conventional radiographic features for enhanced accuracy. Xu et al. (16)studied 170 participants and utilized arterial phase CT radiomics to develop a predictive model, with an AUC of 0.889 in the training cohort, however, the AUC decreased to 0.722 in the validation cohort, indicating a significant decrease in performance, which suggests limitations in model reliability and clinical applicability. Wang et al. (32)integrated clinical features, conventional radiographic features and radiomics features to construct a combined model, achieving AUCs of 0.872 and 0.853 for the training and validation cohorts, respectively. Although their model displayed promising predictive power, it lacked an independent external validation cohort to evaluate its generalizability. In our study, we extracted radiomics features from plain CT images to develop a predictive model for MPP. The model demonstrated promising performance, with AUCs of 0.895 in the training cohort and 0.834 in the internal validation cohort. We also adopted an external validation cohort to validate the performance of the developed models. The performance of the developed models in the external validation cohort remained stable. During the construction of the radiomics model, we found that radiomic features extracted on 3D images played a crucial role. As a representative example of radiomics, voxelbased histogram analysis (VHA) based on 3D images, have been used in identifying early-stage LUAD suitable for sublobar resection (21). In contrast to 2D features, 3D features can identify texture variations and irregular shapes in different regions of the tumor, thereby enhancing the understanding of its biological characteristics. Conventional radiographic features, such as the lobulation sign, spiculation sign, maximum tumor diameter, and the CTR, are all 2D features. This limitation may account for the observation that clinical models utilizing 2D features often exhibit lower predictive performance compared to radiomics models. Additionally, we constructed the nomogram model integrating nodule type, maximum tumor diameter, and the radiomics signature to enhance the predictive accuracy for MPP in patients with LUAD. Notably, the AUCs of the nomogram in the training, internal validation and external validation cohorts were 0.905, 0.850, and 0.817, respectively. Compared with both the clinical and radiomics models, the nomogram exhibited improved predictive performance across all cohorts. The nomogram combines the benefits of both the clinical model and the radiomics model. In clinical practice, the nomogram can be used to evaluate the precise risk of MPP in each LUAD patient on basis of specific clinical indicators, conventional radiographic features and Radscore. This nomogram will aid in the individualized assessment of patient survival risk, providing a reference for clinicians to devise rational and effective treatment strategies.

Our study has several limitations. First, this investigation was retrospective and therefore limited by biases such as incomplete data acquisition and patient selection. Second, the sample size was relatively modest, which may have influenced the generalizability of the findings. Future endeavors will be aimed to increase the sample size and extend the research to multiple centers for enhanced statistical robustness. Third, our study did not utilize more advanced technologies, such as deep learning methods. Consequently, we intend to incorporate deep learning methods in our study as a next step.

## Conclusions

In conclusion, we established a nomogram model based on radiomics and clinical features to distinguish MPP in LUAD patients with a tumor size of ≤2 cm that exhibited good performance. Compared with the clinical model and radiomics model, the nomogram model exhibited a greater level of predictive accuracy, providing a promising method to aid clinicians in developing more rational and efficacious personalized treatment strategies.

## Data Availability

The raw data supporting the conclusions of this article will be made available by the authors, without undue reservation.
