# Supplementary material for: Preoperative CT-based radiomics nomogram to predict the micropapillary pattern in lung adenocarcinoma of size 2 cm or less
Source: Front Oncol. 2025 Jan 7;14:1426284. doi: 10.3389/fonc.2024.1426284 (PMC11752897; doi:10.3389/fonc.2024.1426284)
Supplement: Supplementary file 1 [file DataSheet1.docx]

Supplementary Material

# Supplementary Data

Radscore= 0.2619047619047618 -0.000127 * gradient_firstorder_Kurtosis -0.028781 * lbp_3D_k_gldm_DependenceVariance +0.024759 * lbp_3D_k_glszm_LargeAreaLowGrayLevelEmphasis -0.004627 * lbp_3D_k_glszm_SmallAreaLowGrayLevelEmphasis +0.014582 * lbp_3D_k_ngtdm_Busyness -0.025483 * log_sigma_1_0_mm_3D_firstorder_10Percentile +0.033374 * log_sigma_1_0_mm_3D_glcm_Correlation +0.004060 * log_sigma_2_0_mm_3D_firstorder_Kurtosis -0.022131 * log_sigma_3_0_mm_3D_firstorder_RootMeanSquared -0.032004 * logarithm_glszm_SmallAreaEmphasis +0.048470 * original_firstorder_Kurtosis -0.010323 * square_firstorder_Skewness -0.015594 * square_glrlm_ShortRunLowGrayLevelEmphasis -0.015210 * squareroot_firstorder_Skewness +0.010923 * squareroot_glszm_GrayLevelNonUniformity -0.003672 * squareroot_glszm_ZonePercentage -0.003876 * squareroot_ngtdm_Strength +0.015026 * wavelet_HHL_glcm_Correlation +0.020401 * wavelet_HLH_glszm_SmallAreaHighGrayLevelEmphasis -0.001638 * wavelet_HLL_glrlm_RunVariance -0.008217 * wavelet_LHL_glcm_ClusterShade +0.065241 * wavelet_LLH_glszm_GrayLevelNonUniformity。
