# Supplementary material for: Preoperative CT-based radiomics nomogram to predict the micropapillary pattern in lung adenocarcinoma of size 2 cm or less
Source: Front Oncol. 2025 Jan 7;14:1426284. doi: 10.3389/fonc.2024.1426284 (PMC11752897; doi:10.3389/fonc.2024.1426284)
Supplement: Supplementary file 2 [file DataSheet2.docx]

We have assessed this study using the RQS scale, the scoring criteria and results are as follows. These results will be included in the supplementary materials.

**Radiomics quality score (RQS) scoring:**

**1 Image protocol quality - well-documented image protocols (for example, contrast, slice thickness, energy, etc.) and/or usage of public image protocols allow reproducibility/replicability.**

The CT images acquisition process, which encompasses patient preparation and parameter settings for the CT scan, is extensively described in this study's CT image acquisition section. As a result, this study scored 1 point for this item.

**2 Multiple segmentations - possible actions are: segmentation by different physicians/algorithms/software, perturbing segmentations by (random) noise, segmentation at different breathing cycles. Analyse feature robustness to segmentation variabilities.**

Our study describes the extraction process of tumor segmentation and radiomics features in Tumor Segmentation and Feature Extraction in Materials and methods. A radiologist with over five years of work experience used the ITK-SNAP software （ITK-SNAP 3.8.0, www.itksnap.org）for 3D manual segmentation. Outlining all lesions was reviewed and confirmed by another senior radiologist over five years of work experience. Any deviation is effectively addressed and resolved through thorough discussion. As a result, this study scored 1 point for this item.

**3 Phantom study on all scanners - detect inter-scanner differences and vendor-dependent features. Analyse feature robustness to these sources of variability.**

This study is a dual-center research, where five different models of CT scanners were used to acquire chest CT images. The scanning parameters of each CT machine were kept as consistent or similar as possible. Additionally, we standardized the CT images and resampled them to voxel sizes of 1 mm × 1 mm × 1 mm to reduce the impact of different acquisition parameters. As a result, this study scored 1 point for this item.

**4 Imaging at multiple time points - collect images of individuals at additional time points. Analyse feature robustness to temporal variabilities (for example, organ movement, organ expansion/shrinkage).**

This study scored 0 points for this item.

**5 Feature reduction or adjustment for multiple testing - decreases the risk of overfitting. Overfitting is inevitable if the number of features exceeds the number of samples. Consider feature robustness when selecting features.**

The feature selection and model construction process were described in the "Materials and Methods" section of this study. The AUC values in the training cohorts, internal validation cohort and external validation cohort confirmed the model did not overfit. As a result, this study scored 3 points for this item.

**6 Multivariable analysis with non radiomics features (for example, EGFR mutation) - is expected to provide a more holistic model. Permits correlating/inferencing between radiomics and non radiomics features.**

This study combined radiomics features with radiological features to construct a nomogram. As a result, this study scored 1 point for this item.

**7 Detect and discuss biological correlates - demonstration of phenotypic differences (possibly associated with underlying gene–protein expression patterns) deepens understanding of radiomics and biology.**

This study scored 0 points for this item.

**8 Cut-off analyses - determine risk groups by either the median, a previously published cut-off or report a continuous risk variable. Reduces the risk of reporting overly optimistic results.**

This study scored 0 points for this item.

**9 Discrimination statistics - report discrimination statistics (for example, C-statistic, ROC curve, AUC) and their statistical significance (for example, p-values, confidence intervals). One can also apply resampling method (for example, bootstrapping, cross-validation).**

In our study, ROC curves were plotted for each model, and the AUC values of each model were calculated. The DeLong test was used to assess the differences between the ROC curves. As a result, this study scored 1 point for this item.

**10 Calibration statistics - report calibration statistics (for example, Calibration-in-the-large/slope, calibration plots) and their statistical significance (for example, P-values, confidence intervals). One can also apply resampling method (for example, bootstrapping, cross-validation).**

In this study, the calibration curves were plotted. As a result, this study scored 1 point for this item.

**11 Prospective study registered in a trial database - provides the highest level of evidence supporting the clinical validity and usefulness of the radiomics biomarker.**

This study scored 0 points for this item.

**12 Validation - the validation is performed without retraining and without adaptation of the cut-off value, provides crucial information with regard to credible clinical performance.**

The models were validated using a dataset from two hospitals.As a result, this study scored 3 points for this item.

**13 Comparison to 'gold standard' - assess the extent to which the model agrees with/is superior to the current 'gold standard' method (for example, TNM-staging for survival prediction). This comparison shows the added value of radiomics.**

The model was compared with pathological results, which served as the gold standard. As a result, this study scored 2 points for this item.

**14 Potential clinical utility - report on the current and potential application of the model in a clinical setting (for example, decision curve analysis).**

Decision curve analysis (DCA) were plotted to evaluate the model performance and clinical application. This study scored 2 points for this item.

**15 Cost-effectiveness analysis - report on the cost-effectiveness of the clinical application (for example, QALYs generated).**

This study scored 0 points for this item.

**16 Open science and data - make code and data publicly available. Open science facilitates knowledge transfer and reproducibility of the study.**

The datasets analyzed during the current study can be found in the supplementary material.Further inquiries can be directed to the corresponding authors.As a result, this study scored 1 point for this item.


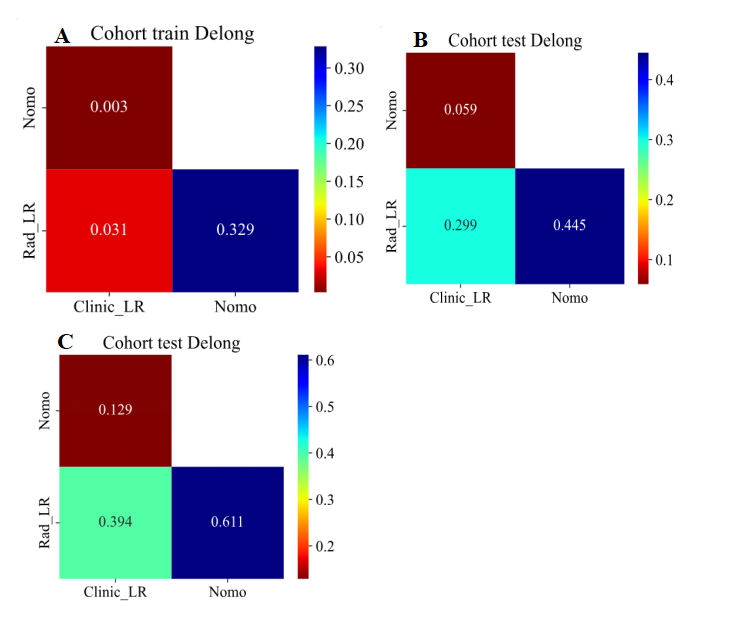


**Supplementary figure 1** | The Delong test in the training cohort (A), internal validation cohort (B) and external validation cohort (C).

**Supplementary** Table 1. The *P-*value of Delong test in three cohorts

| Cohort | Nomogram vs Clinical | Nomogram vs radiomics | radiomics vs Clinical |
| --- | --- | --- | --- |
| Training cohort | 0.003 | 0.329 | 0.031 |
| Internal validation cohort | 0.059 | 0.445 | 0.299 |
| External validation cohort | 0.129 | 0.611 | 0.394 |

**The code for DeLong's test is as follows.**

**from onekey_algo.custom.components.delong import delong_roc_test**

**from onekey_algo.custom.components.comp1 import draw_matrix**

**delong = []**

**delong_columns = []**

**this_delong = []**

**plt.figure(figsize=(5, 4))**

**cm = np.zeros((len(model_names), len(model_names)))**

**for i, mni in enumerate(model_names):**

**for j, mnj in enumerate(model_names):**

**if i <= j:**

**cm[i][j] = np.nan**

**else:**

**cm[i][j] = delong_roc_test(ALL_results[task], ALL_results[mni], ALL_results[mnj])[0][0]**

**cm = pd.DataFrame(cm[1:, :-1], index=model_names[1:], columns=model_names[:-1])**

**draw_matrix(cm, annot=True, cmap='jet_r', cbar=True)**

**plt.title(f'Cohort {subset} Delong')**

**plt.savefig(f'img/compare_delong_each_cohort_{subset}.svg', bbox_inches = 'tight')**

**plt.show()**
